# Ultrasound for Neurogenic Thoracic Outlet Obstruction Remains Theoretical

**DOI:** 10.3390/diagnostics10040205

**Published:** 2020-04-07

**Authors:** Mitchell P. Wilson, Gavin Low, Prayash Katlariwala, Line Jacques, Andrew S. Jack

**Affiliations:** 1 Department of Radiology and Diagnostic Imaging, University of Alberta, 2B2.41 WMC, 8440-112 Street NW, Edmonton, AB T6G 2B7, Canada; timgy@yahoo.com (G.L.); prayashvk@gmail.com (P.K.); 2 Department of Neurosurgery, University of California, San Francisco (UCSF), 400 Parnassus Ave, San Francisco, CA 94143, USA; line.jacques@ucsf.edu (L.J.); asjack@ualberta.ca (A.S.J.)

We enjoyed reading Povleson et al.’s review entitled “Diagnostic thoracic outlet syndrome: current approaches and future directions” [1]. The authors performed a thorough review of current and emerging investigations available for the diagnosis of thoracic outlet syndrome (TOS). We were particularly interested in the authors’ evaluation of neurogenic TOS, which is known to be difficult on account of the brachial plexus branching patterns and various constellations of presenting symptomatology. Clinical testing has been shown to lack sufficient sensitivity and specificity for neurogenic TOS [2].

In the Povleson et al. review, brachial plexus ultrasound is identified as an emerging technology for neurogenic TOS. The authors highlight the relative low cost and availability as benefits of ultrasound over MRI (magnetic resonance imaging) for the investigation. To support this argument, the authors cite one study identifying an increased rate of neurogenic symptoms in patients with an atypical branching anatomy of the brachial plexus compared with those with a normal branching anatomy in a small feasibility study with a poor methodology ([4/8] 50% versus [5/35] 14%) [3].

To further explore the potential utility of ultrasound for neurogenic TOS, we attempted a comparative systematic review and meta-analysis assessing the diagnostic accuracy of both ultrasound and MRI evaluated against a surgical reference standard (PROSPERO Registration ID: 168479). All relevant articles up to 12 January 2020 in MEDLINE, EMBASE, Scopus, and the Cochrane Library were evaluated. A total of 178 potentially relevant articles were identified after duplicate removal. However, following the title and abstract review and subsequent full text review, no articles evaluating ultrasound for neurogenic TOS were available for inclusion. The results of this systematic review highlight the lack of available evidence to support the assertion that ultrasound could be used as a diagnostic test for neurogenic TOS. At present, the utility of ultrasound for this clinical scenario remains theoretical rather than emerging. Future studies evaluating the diagnostic accuracy of ultrasound for neurogenic TOS with a rigorous methodological standard including a reference standard that can minimize both selection bias and verification bias (such as electrodiagnostic studies and/or a combination of investigations) and reasonable sample size are needed.
